# Left bundle, right diagnosis

**DOI:** 10.1007/s12471-019-1256-z

**Published:** 2019-03-01

**Authors:** A. E. Schaafsma, C. Coolsma, H. Lameijer

**Affiliations:** Department of Emergency Medicine, Medical Centre Leeuwarden, Leeuwarden, The Netherlands

## Answer

**Fig. 1:**
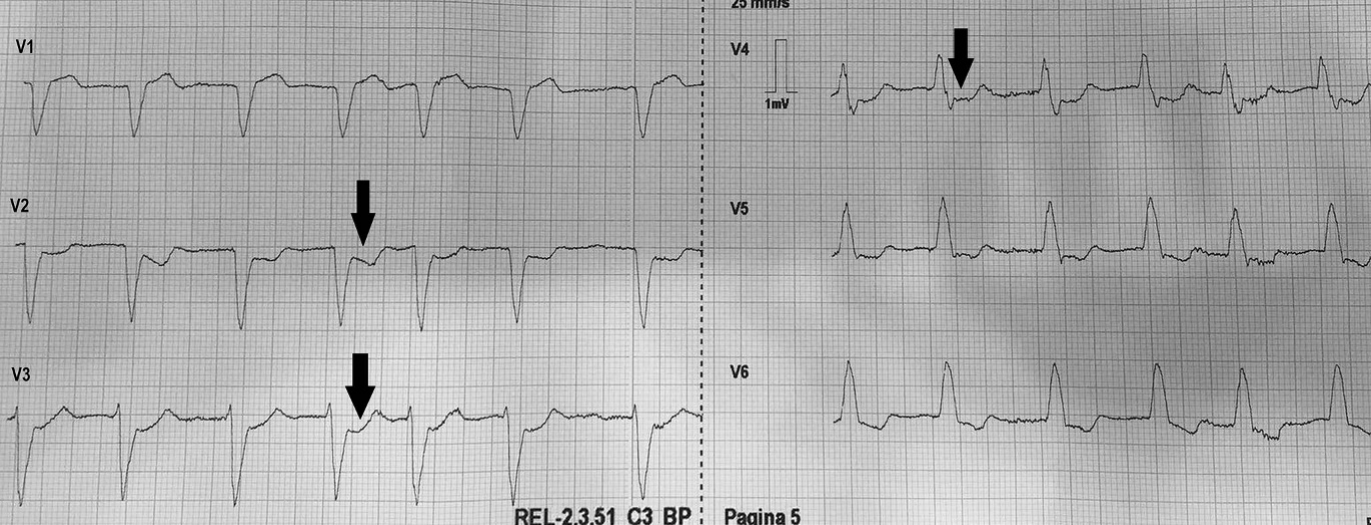
The ECG as in Fig. 1 of the rhythm puzzle question, showing LBBB with ST depression in V2–V4 in the anterior leads (*arrows*), which is a Sgarbossa criterion. *ECG* electrocardiogram, *LBBB* left bundle branch block

The partial electrocardiogram (Fig. 1) shows a left bundle branch block (LBBB), possibly caused by acute coronary syndrome (ACS). However, it can also be pre-existent, or based on other cardiac disease. To evaluate ACS in patients with LBBB, the Sgarbossa criteria should be used [1]. Sgarbossa criteria comprise three electrocardiographic criteria for diagnosis of ACS in patients with LBBB: ST-segment elevation of 1 mm or more that is concordant with the QRS complex; ST-segment depression of 1 mm or more in leads V1, V2, or V3; and ST-segment elevation of 5 mm or more that is disconcordant with (in the direction opposite) the QRS complex. Each criterion increases the chance of ACS in patients presenting with LBBB. Smith et al. modified these criteria by replacing the third criterion with an ST/S ratio less than −0.25, improving the diagnosis of STEMI in these patients [2].

In this electrocardiogram, LBBB with ST depression in V2–V4 in the anterior leads is observed. This observation, in combination with the patient’s complaints, is sufficient reason to consider a STEMI equivalent. The patient was treated with heparin 5,000 IE (intravenous route), acetylsalicylic acid 500 mg and ticagrelor 180 mg and nitroglycerine (intravenous route). Emergency coronary angiography showed a significant stenosis of both the left main and right coronary artery, not accessible for percutaneous coronary intervention. The patient was treated with emergency coronary artery bypass grafting.
